# Mst1-FoxO Signaling Protects Naïve T Lymphocytes from Cellular Oxidative Stress in Mice

**DOI:** 10.1371/journal.pone.0008011

**Published:** 2009-11-24

**Authors:** Juhyun Choi, Sangphil Oh, Dongjun Lee, Hyun Jung Oh, Jik Young Park, Sean Bong Lee, Dae-Sik Lim

**Affiliations:** 1 National Research Laboratory of Molecular Genetics, Department of Biological Sciences, Biomedical Research Center, Korea Advanced Institute of Science and Technology, Daejeon, South Korea; 2 Genetics of Development and Disease Branch, National Institute of Diabetes & Digestive & Kidney Diseases, National Institutes of Health, Bethesda, Maryland, United States of America; University of Washington, United States of America

## Abstract

**Background:**

The Ste-20 family kinase Hippo restricts cell proliferation and promotes apoptosis for proper organ development in *Drosophila*. In *C. elegans*, Hippo homolog also regulates longevity. The mammalian Ste20-like protein kinase, Mst1, plays a role in apoptosis induced by various types of apoptotic stress. Mst1 also regulates peripheral naïve T cell trafficking and proliferation in mice. However, its functions in mammals are not fully understood.

**Methodology/Principal Findings:**

Here, we report that the Mst1-FoxO signaling pathway plays a crucial role in survival, but not apoptosis, of naïve T cells. In *Mst1^−/−^* mice, peripheral T cells showed impaired FoxO1/3 activation and decreased FoxO protein levels. Consistently, the FoxO targets, Sod2 and catalase, were significantly down-regulated in *Mst1^−/−^* T cells, thereby resulting in elevated levels of intracellular reactive oxygen species (ROS) and induction of apoptosis. Expression of constitutively active FoxO3a restored *Mst1^−/−^* T cell survival. Crossing Mst1 transgenic mice (*Mst1* Tg) with *Mst1^−/−^* mice reduced ROS levels and restored normal numbers of peripheral naïve T cells in *Mst1* Tg;*Mst1^−/−^* progeny. Interestingly, peripheral T cells from *Mst1^−/−^* mice were hypersensitive to γ-irradiation and paraquat-induced oxidative stresses, whereas those from *Mst1* Tg mice were resistant.

**Conclusions/Significance:**

These data support the hypothesis that tolerance to increased levels of intracellular ROS provided by the Mst1-FoxOs signaling pathway is crucial for the maintenance of naïve T cell homeostasis in the periphery.

## Introduction

Maintenance of T cell homeostasis is critical for normal functioning of the immune system. Fas, TNF, and ROS effectively promote the elimination of antigen-specific activated T cells and limit autoimmunity [1], [2]. Intracellular redox status is a physiological regulator of T cell activation and apoptosis during normal T cell development in the thymus, and also regulates the immune response in peripheral lymphoid organs [3]. Recent studies have shown that the transcription factor, FoxO1, is critical for maintaining naïve T cells in peripheral lymphoid organs by virtue of its regulation of L-selectin (CD62L), CCR7 and interleukin 7 receptor α (IL-7Rα/CD127) expression [4], [5]. However, naïve T cell homeostatic mechanisms in protective immunity are not fully understood.

In mammals, Mst1 (mammalian sterile 20-like 1) kinase, which is a key component of the “Hippo” signaling pathway, has been implicated in regulating the cell cycle, apoptosis and cellular responses to oxidative stress [6]. Recently, Mst1-deficient (*Mst1^−/−^*) mice were shown to possess decreased numbers of peripheral T cells, mostly naïve T cells; those cells that remained showed increased proliferation after T cell receptor (TCR) ligation in vitro and impaired lymphocyte homing to the spleen and lymph nodes [7], [8]. Additionally, defective egress of mature thymocytes from *Mst1^−/−^* thymus were also reported [7], [9]. The Rassf adapter protein, Rapl (also named Nore1b), is known to activate Mst1 by regulating its localization and kinase activity during lymphocyte migration to lymphoid organs [10]. These observations suggest that the Rapl-Mst1 pathway can modulate cell proliferation and T-cell migration [7], [8]. Consistent with these recent findings, we observed a similar phenotype in *Mst1^−/−^* mice. In addition, we identified a new and important role for the Mst1-FoxO signaling pathway in regulating the homeostasis of peripheral naïve T cells.

## Results and Discussion

### Mst1 Deficiency Causes Systemic Lymphopenia

To define the physiological functions of Mst1 in higher organisms, we generated mice lacking Mst1. Lymphoid tissues from *Mst1^−/−^* mice displayed a paucity of T and B cells in the spleen and reduced numbers of T cells in lymph nodes; in particular, a large number of cells in white pulp and T cell zones appeared to be absent (Figure S1A and B). We confirmed that the numbers of T cells in spleen, lymph nodes, and peripheral blood *of Mst1^+/+^* and *Mst1^+/−^* mice were not different, indicating the haplosufficiency of Mst1 with respect to leukocyte generation (Figure S2). *Mst1^+/+^* or *Mst1^+/−^* mice were thus used as controls. Consistent with recent findings from Mst1-deficient mice [7]-[9], we also observed the reduced numbers of both CD4^+^ and CD8^+^ peripheral T cells in *Mst1^−/−^* mice (Figure S2A). Most of the absent T cells in peripheral blood, spleen, and lymph nodes were naïve T cells, not effector/memory T cells (Figure S2B). Although the ratio of single-positive CD4 and CD8 cells appeared to be slightly increased in the thymus from *Mst1^−/−^* mice (Figure S1E), the thymic architecture and the actual numbers of these cells were similar in *Mst1^+/+^*, *Mst1^+/−^*, and *Mst1^−/−^* mice (Figure S1C and D). The thymic development of *Mst1^−/−^* T cells bearing TCRs was also normal, as was their environmental epithelium (unpublished data). Thus, we concluded that the reduced number of peripheral naïve T cells from *Mst1^−/−^* mice does not result from impaired T cell development or thymic development.

### Mst1 Deficiency Causes Naïve T Cell Death

**Figure 1:**
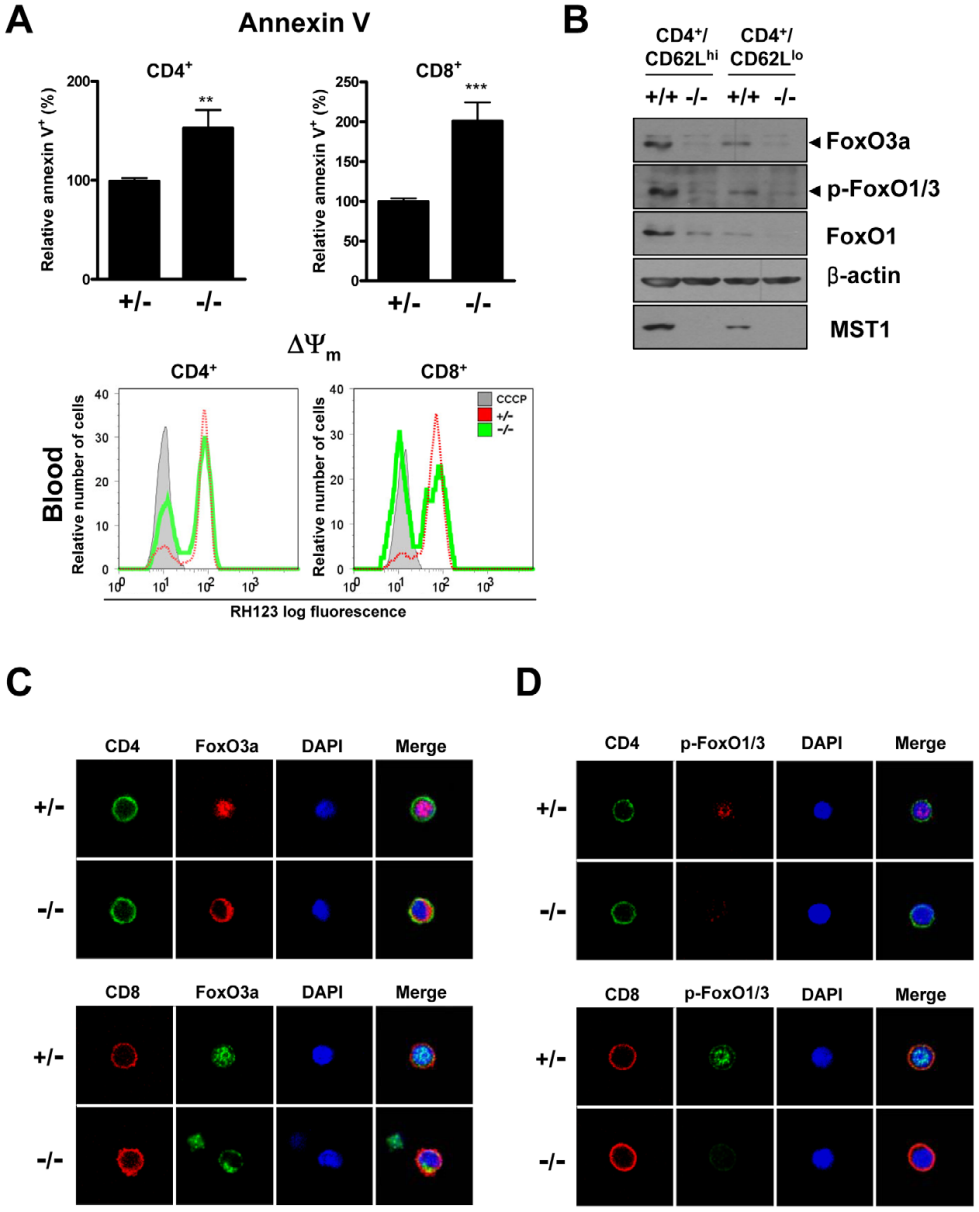
Increased apoptosis and dysregulation of FoxO1/3 proteins in peripheral T cells from *Mst1* ^−/−^ mice. (A) *Top*: Apoptotic cell death of peripheral blood T cells from *Mst1^+/−^* and *Mst1* **^−/−^** mice (n = 13) was detected. The percentage of Annexin V-positive cells was determined by normalization to the percentage of *Mst1^+/−^* Annexin V-positive cells (defined as 100%). Error bars indicate SEM. **, *p*<0.01; ***, *p*<0.001. *Bottom*: Mitochondrial transmembrane potential (ψ_m_) of CD4^+^ and CD8^+^ T cells in peripheral blood from *Mst1^+/−^* and *Mst1* **^−/−^** mice was determined by staining with Rhodamine 123. CCCP-treated cells were used as a control (grey). FACS profiles shown are representative of three independent experiments. (B) FoxO1/3 protein levels and phosphorylation were decreased in *Mst1^−/−^* T cells. CD4^+^/CD62L^hi^ or CD4^+^/CD62L^lo^ T cells from spleens were purified by MACS. Cell lysates were then analyzed by immunoblotting. (C) Immunostaining of FoxO3a in peripheral blood T cells from *Mst1^+/−^* and *Mst1* **^−/−^** mice. Lymphocytes were stained for CD4 (green) and FoxO3a (red), or CD8 (red) and FoxO3a (green); nuclei were stained with DAPI (blue). (D) Immunostaining of p-FoxO1/3 in peripheral blood T cells from *Mst1^+/−^* and *Mst1* **^−/−^** mice. Lymphocytes were stained for CD4 (green) and p-FoxO1/3 (red), or CD8 (red) and p-FoxO1/3 (green); nuclei were stained with DAPI (blue).

Mst1 promotes cellular oxidative stress and/or growth factor deprivation-induced apoptosis in neurons [6], [11]. Therefore, we first tested whether Mst1 deficiency affected the survival of T cells. Interestingly, many of the peripheral CD4^+^ and CD8^+^ T cells in *Mst1^−/−^* mice rapidly underwent apoptosis (Figure 1A, top). There was also a loss of mitochondrial transmembrane potential (ψ_m_) in these *Mst1^−/−^* peripheral CD4^+^ and CD8^+^ T cells (Figure 1A, bottom), confirming apoptosis. Thus, spontaneous cell death of peripheral *Mst1^−/−^* T cells might be responsible for the reduced numbers of peripheral T cells in *Mst1^−/−^* mice.

To explore the basis for this reduced cellularity and/or apoptosis of peripheral T cells in *Mst1^−/−^* mice, we first crossed Fas- and FasL-defective mice (lpr and gld, respectively) with *Mst1^−/−^* mice. Neither *Mst1^−/−^;Fas^lpr/lpr^* nor *Mst1^−/−^;FasL^gld/gld^* mice exhibited a restoration of peripheral T cells to normal levels (Figure S3), indicating that the apoptosis of *Mst1^−/−^* T cells was independent of Fas and FasL. An examination of serum cytokines and chemokines revealed no significant differences between *Mst1^+/−^* and *Mst1^−/−^* mice. Notably, the levels of IL-7, which is critical for the maintenance of both naïve and memory T cell homeostasis [12], and tumor necrosis factor (TNF), which is associated with apoptosis of activated T cells [13], were similar in the sera of *Mst1^+/−^* and *Mst1^−/−^* mice (Figure S4).

### FoxO Pproteins Are Inactivated in *Mst1^−/−^* Peripheral T Cells

Mst1 phosphorylates and activates FoxO3a on serine 207, promoting cellular oxidative stress-induced apoptosis in neurons and longevity in *Caenorhabditis elegans* [6]. Phosphorylation of FoxO1 on serine 212 by Mst1 also regulates neuronal cell death under conditions of growth-factor deprivation [11]. Akt and Mst1 exhibit mutually antagonistic functions, and FoxO proteins can be inactivated by Akt [14]–[16]. On the basis of these reports, we examined the expression levels of FoxO1/3 proteins in peripheral CD4^+^, CD8^+^, naïve (CD62L^hi^CD44^lo^), and effector/memory (CD62L^lo^CD44^hi^) T cells. Interestingly, *Mst1* **^−/−^** T cells displayed reduced levels of FoxO3a and FoxO1 proteins, and the phosphorylated form of FoxO1/3 (Figures 1B and S5). Thus, we hypothesized that Mst1 is required for maintaining normal levels of FoxO1/3 proteins, and further postulated that inactivation of these proteins might disrupt peripheral naïve T cell homeostasis.

In primary neurons, FoxO3a subcellular localization is reciprocally regulated by MST1, which promotes nuclear localization by phosphorylating FoxO3a on serine 207 in response to hydrogen peroxide (H_2_O_2_), and by Akt, which phosphorylates FoxO proteins and induces their translocation to the cytoplasm where they are subsequently degraded [6], [16]. Using immunostaining to examine the subcellular localization of FoxO3a in peripheral T cells, we found that FoxO3a was primarily localized to the nucleus in *Mst1^+/−^* T cells (∼85%). By contrast, the majority of FoxO3a was localized in the cytoplasm of peripheral *Mst1^−/−^* T cells (∼65%), and its staining intensity was significantly weaker than in *Mst1^+/−^* T cells (Figure 1C). Consistent with this observation, FoxO1/3 was detected in an activated, phosphorylated state in a significant proportion (∼37%) of *Mst1^+/−^* peripheral T cell nuclei, whereas negligible numbers of similarly stained T cells were detected in *Mst1^−/−^* mice (Figure 1D). We were unable to specifically detect FoxO1 protein because FoxO1 antibodies suitable for immunostaining are unavailable. These data show that Mst1 modulates FoxO1/3 nuclear localization and activation in peripheral T cells.

### Expression of Sod2 and Catalase Are Significantly Down-Regulated in *Mst1^−/−^* Peripheral T Cells

**Figure 2:**
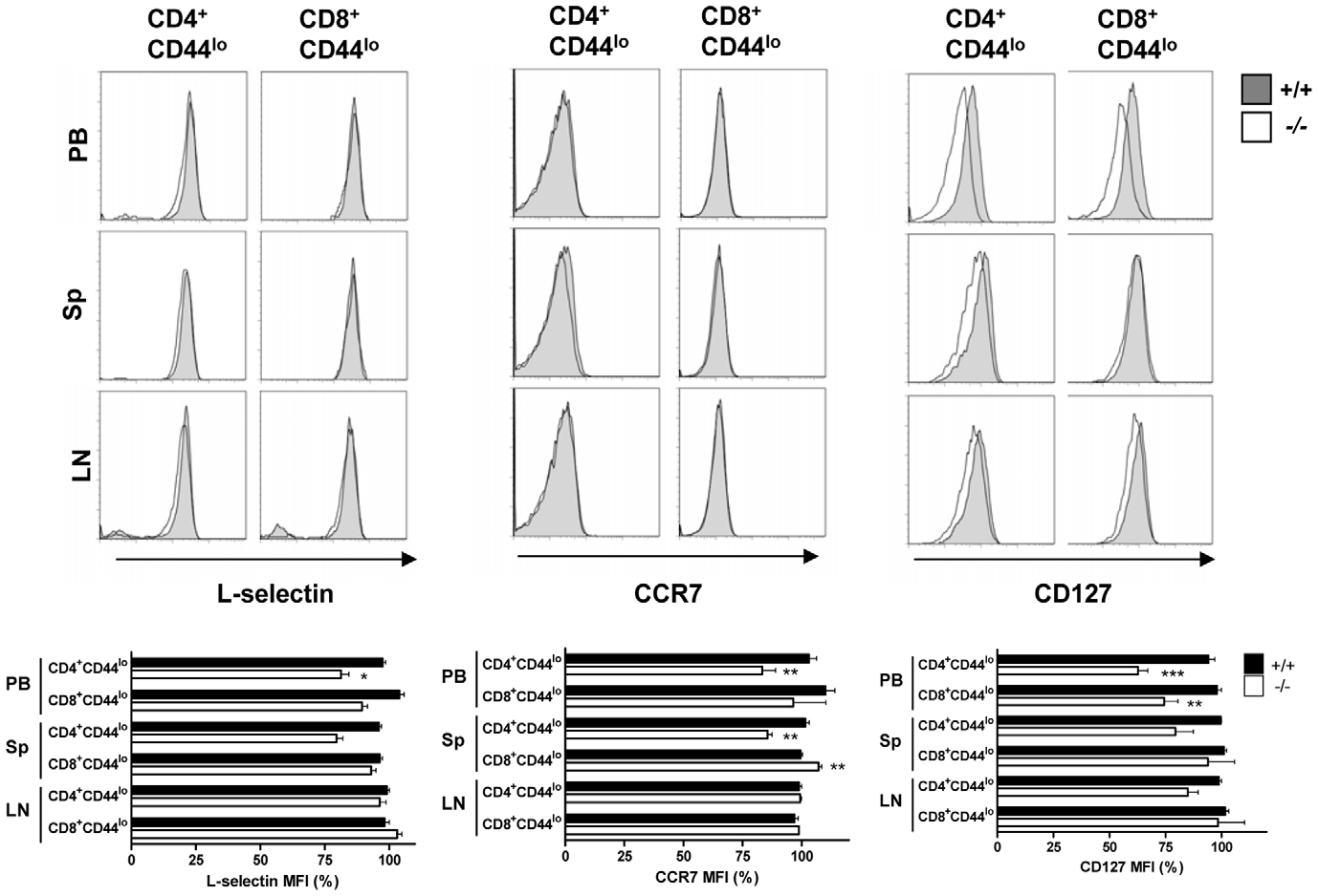
Surface expression of L-selectin, CCR7, and IL-7Rα in *Mst1^−/−^* peripheral T cells. L-selectin (n≥6), CCR7 (n≥3), and CD127 (n≥3) expression on naive T cells from peripheral blood (PB), spleen (Sp), lymph node (LN) were quantified by FACS. MFI, mean fluorescent intensity. *, *p*<0.05; **, *p*<0.01; ***, *p*<0.001.

Recent reports have shown that FoxO1 deletion in T cells induces a reduction in the number of peripheral naïve T cells due to a defect in the expression of lymphocyte-trafficking molecules (L-selectin and CCR7) and IL-7Rα [4], [5], indicating a critical role for FoxO1 in homeostasis of naïve T cells. On the basis of these reports and our observation that FoxO1/3 expression was reduced in *Mst1^−/−^* T cells, we examined the expression of L-selectin, CCR7 and IL-7Rα in *Mst1^−/−^* naïve T cells. Although IL-7Rα expression was slightly reduced in peripheral blood, the cell surface levels of these proteins did not show statistically significant difference between *Mst1^+/+^* and *Mst1^−/−^* naïve T cells (Figure 2). Thus, it is likely that a reduction in FoxO1 protein levels, unlike ablation of the *FoxO1* gene, does not significantly affect the expression of these molecules in *Mst1^−/−^* mice.

**Figure 3:**
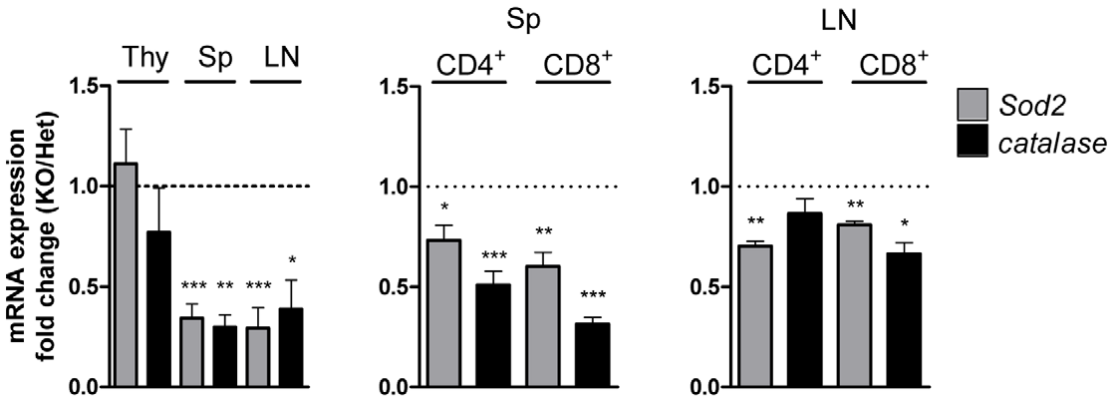
Dysregulation of FoxO1/3 results in down-regulation of Sod2 and catalase. Quantitative RT-PCR analysis of Sod2 and catalase mRNA expression in lymphocytes from thymus, spleen, and inguinal lymph nodes of *Mst1^+/−^* and *Mst1^−/−^* mice. Lymphocytes from spleen and lymph nodes were further purified into CD4^+^ and CD8^+^ T cells by MACS. Target mRNA expression levels were normalized to endogenous β*-*actin. Fold changes were calculated by measuring *Mst1^−/−^*/*Mst1^+/−^* ratios. Data are pooled from three independent experiments of triplicate. Error bars indicate SEM. *, *p*<0.05; **, *p*<0.01; ***, *p*<0.001.

FoxO family factors stimulate transcription of *Sod2* and catalase (*Cat*), which are important in reducing cellular oxidative stress [17], [18]. Thus, we then examined the expression levels of these FoxO targets in T cells. Consistent with a role for the Mst1-FoxO pathway in regulating *Sod2* and *Cat* expression, quantitative PCR analyses showed that Sod2 and catalase mRNA levels were significantly decreased in T cells derived from *Mst1^−/−^* spleens and lymph node compared to those from *Mst1^+/−^* mice (Figure 3). Consistent with reduced mRNA levels, their protein levels were also reduced in *Mst1^−/−^* T cells (Figure S5) Thus, in the absence of Mst1, reducing and/or inactivating FoxO1/3 could result in decreases in Sod*2* and catalase expression in CD4^+^ and CD8^+^ T cells.

### Increased ROS in *Mst1^−/−^* Peripheral T Cells Induces Apoptosis

**Figure 4:**
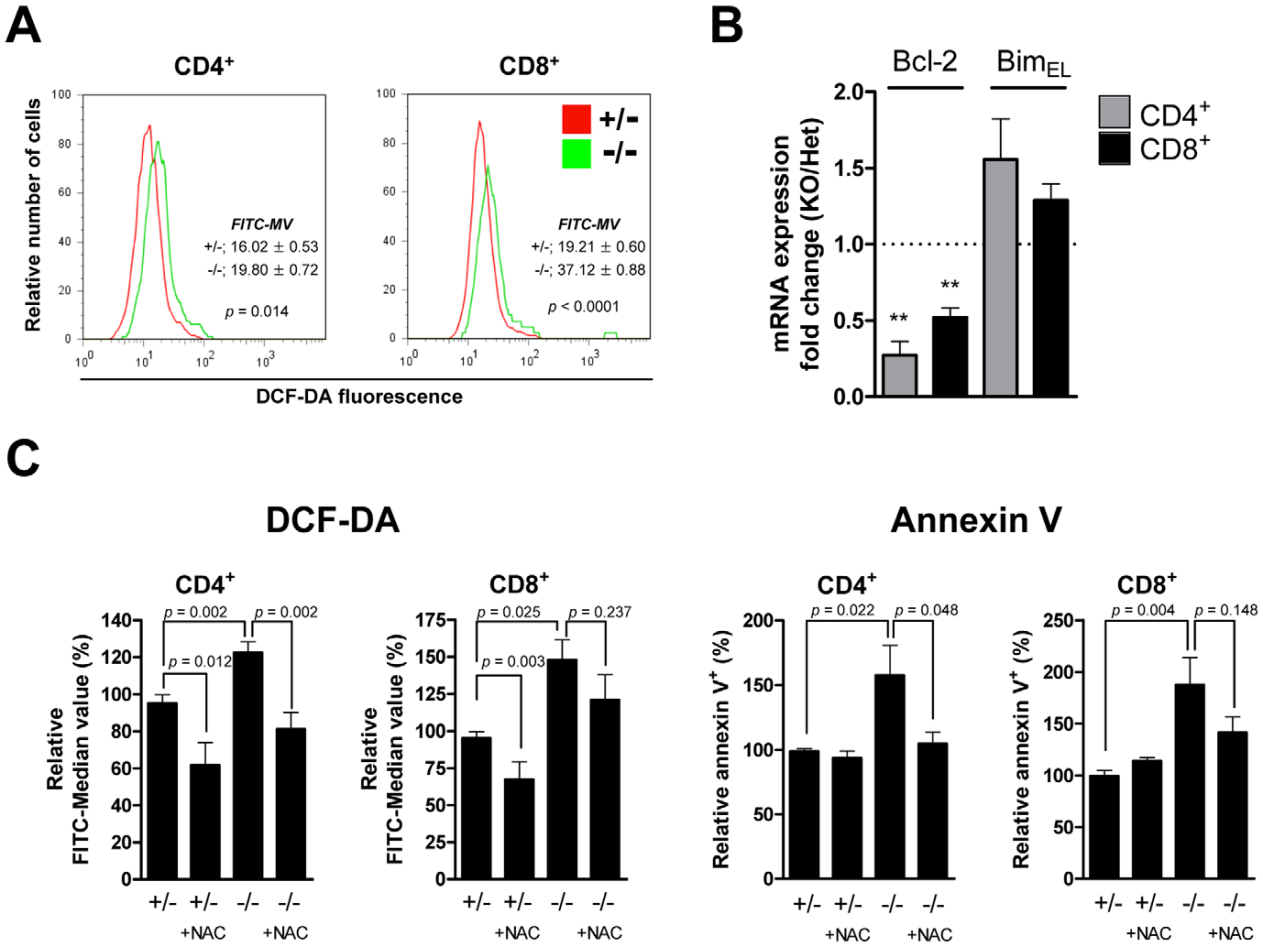
Mst1 is important in ROS regulation and peripheral T cell survival. (A) Intracellular ROS levels in peripheral blood CD4^+^ and CD8^+^ T cells from *Mst1^+/−^* and *Mst1^−/−^* mice were detected by staining with DCF-DA. Data are representative of three independent experiments. (B) Quantitative RT-PCR analysis of Bcl-2 and Bim_EL_ mRNA expression in splenic CD4^+^ and CD8^+^ T cells (n = 5 for Bcl-2 and n = 3 for Bim_EL_). **, *p*<0.01. (C) ROS levels (left) and apoptotic cell death (right) in peripheral blood T cells from *Mst1^+/−^* and *Mst1* **^−/−^** mice (n≥5 for each) with or without NAC (10 ng/ml) treatment were determined. Relative FITC-median values of DCF-DA fluorescence were analyzed for CD4^+^ and CD8^+^ populations. The percentage of Annexin V-positive cells was determined by normalization to the percentage of *Mst1^+/−^* Annexin V-positive cells (defined as 100%). Error bars indicate SEM.

Because Sod2 and catalase were down-regulated in *Mst1^−/−^* T cells, we tested whether the levels of intracellular ROS were increased in T cells from *Mst1^−/−^* mice, and thus might account for the increase in apoptosis. Although the overall levels of intracellular ROS in thymic T cells were similar in *Mst1^+/−^* and *Mst1^−/−^* mice, intracellular ROS levels increased gradually during the progression from double-positive to single-positive T cells in the thymus (unpublished data). Interestingly, the peripheral CD4^+^ and CD8^+^ T cells in *Mst1^−/−^* mice showed elevated ROS levels compared to *Mst1^+/−^* mice (Figure 4A). Increased ROS can produce DNA double-strand breaks (DSBs) in mammalian cells [19]. We therefore examined γH2AX, an established measure of DSBs, in T cells. As expected, CD4^+^ and CD8^+^ T cells from *Mst1^−/−^* mice showed an increase in γH2AX signals compared with *Mst1^+/−^* T cells (Figure S6), indicating the presence of DSBs. Intracellular ROS can regulate Bcl-2 levels in activated T cells [2], and the Bim/Bcl-2 balance is critical for maintaining naïve and memory T cell homeostasis [20]. Therefore, we also investigated Bcl-2 involvement in the T cell death associated with increased ROS in *Mst1^−/−^* mice. Bcl-2 mRNA expression was decreased about 2- to 3-fold in *Mst1^−/−^* compared with *Mst1^+/−^* T cells; however, the expression level of *Bim_EL_*, an antagonist of Bcl-2, in *Mst1^−/−^* T cells was increased compared with controls (Figure 4B). Thus, elevated intracellular ROS in *Mst1^−/−^* T cells ultimately decreased Bcl-2 levels, creating a disruption in the balance between Bcl-2 and Bim_EL_ that might contribute to the apoptosis of peripheral *Mst1^−/−^* T cells.

To determine whether the increased apoptosis of *Mst1^−/−^* T cells was primarily due to ROS, we incubated *Mst1^−/−^* T cells from peripheral blood with the antioxidant, N-acetyl-L-cysteine (NAC). At an NAC concentration of 10 ng/ml, intracellular ROS was reduced (Figure 4C, left) and apoptosis was decreased in *Mst1^−/−^* CD4^+^ and CD8^+^ T cells (Figure 4C, right), suggesting that the elevated levels of ROS in peripheral *Mst1^−/−^* T cells resulted in cell death. Although NAC treated *Mst1^+/−^* T cells had lower ROS levels than untreated cells, apoptosis in these cells was not affected by NAC treatment. Although NAC treated Mst1*^−/−^* CD8^+^ T cells have modestly lower levels of ROS compared with untreated Mst1*^−/−^* CD8^+^ T cells, it is also noted that ROS levels in NAC treated *Mst1^−/−^* CD8^+^ T cells were not reduced as much as in CD4^+^ T cells. These differences in response to NAC treatment between CD4^+^ and CD8^+^ T cells may account for smaller change in *Mst1^−/−^* CD8^+^ T cell death compared to CD4^+^ T cell death. Taken together, these results suggest that Mst1-deficient naïve T cells fail to activate FoxO proteins, resulting in a failure to induce Sod2 and catalase expression, the accumulation of intracellular ROS, and increased apoptosis in single-positive T cells.

**Figure 5:**
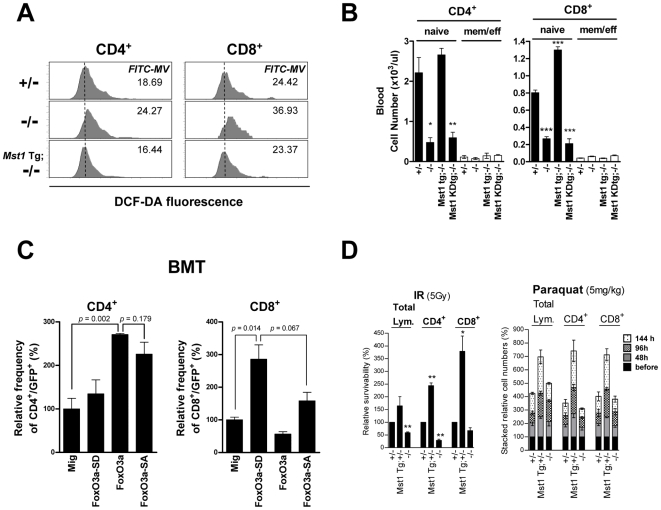
Expression of Mst1 and FoxO3a-SD rescues the peripheral T cell phenotypes of *Mst1^−/−^* mice. (A) Flow cytometric analysis of ROS levels in peripheral blood T lymphocytes from *Mst1^+/−^*, *Mst1^−/−^*, and *Mst1* Tg;*Mst1^−/−^* mice. FITC-median values (FITC-MV) of DCF-DA fluorescence are indicated. Data are representative of three independent experiments. (B) Naïve and effector/memory T cell subsets of peripheral blood from *Mst1^+/−^*, *Mst1^−/−^*, *Mst1* Tg;*Mst1^−/−^*, and *Mst1*KD Tg;*Mst1^−/−^* mice (n≥3) were quantified by FACS. *, *p*<0.05; **, *p*<0.01; ***, *p*<0.001, compared with *Mst1^+/−^* lymphocytes. (C) Bone marrow cells from *Mst1^−/−^* mice were transduced with Mig, Mig-FoxO3a, Mig-FoxO3a-SD, or Mig-FoxO3a-SA retroviral constructs, and 3∼6×10^5^ GFP-positive cells were intravenously injected into irradiated normal C57BL/6J mice. After 4 weeks, peripheral blood GFP^+^CD4^+^ and GFP^+^CD8^+^ T cells were quantified by FACS (n = 5), and the values were expressed as a percentage of GFP-positive lymphocytes in each group. (D) Relative numbers of surviving cells in peripheral blood from *Mst1^+/−^*, *Mst1* Tg;*Mst1* **^+/−^**, and *Mst1* **^−/−^** mice after irradiation or paraquat injection. *Left:* Mice were whole-body irradiated at a dose of 5 Gy, and tail blood was collected before and 72 h after irradiation (n = 3). Number of surviving T cells after irradiation was divided by the pre-irradiation number for each genotype and expressed relative to the *Mst1^+/−^* T-cell survival fraction. *, *p*<0.05; **, *p*<0.01. *Right:* Paraquat (5 mg/kg) was intraperitoneally injected into mice (n = 3) and tail blood was collected before, 48 h, 96 h, and 144 h after injection. The number of surviving T cells was expressed as a fraction of cell numbers before paraquat injection for each genotype. Error bars indicate SEM.

We next generated Mst1 wild-type transgenic (*Mst1* Tg) and kinase-dead transgenic (*Mst1*KD Tg) mice, then tested whether the observed defects were corrected in the *Mst1* Tg;*Mst1* **^−/−^** or *Mst1*KD Tg;*Mst1^−/−^* progeny. In *Mst1* Tg;*Mst1* **^−/−^** mice, intracellular ROS levels in CD4^+^ and CD8^+^ T cells were decreased to levels comparable to those found in *Mst1^+/−^* peripheral T cells (Figure 5A), and the numbers of peripheral naïve T cells were recovered to an even greater extent in *Mst1* Tg;*Mst1* **^−/−^** mice (Figure 5B). In *Mst1* Tg;*Mst1* ***^−/−^*** mice, FoxO3a, Sod2, and catalase protein levels in T cells were also recovered to levels comparable to those in *Mst1^+/−^* T cells (Figure S5). Notably, expression of kinase-dead Mst1 in *Mst1*KD Tg;*Mst1^−/−^* mice failed to restore normal numbers of peripheral blood T cells (Figure 5B), indicating that Mst1 kinase activity toward FoxO1/3 is critical for peripheral T cell homeostasis.

### Expression of FoxO3a-S207D Rescues the Mst1-Null Phenotype

To determine whether Mst1-mediated phosphorylation of FoxO is sufficient to promote the neutralization of elevated intracellular ROS in peripheral T cells, we infected *Mst1* **^−/−^** bone marrow cells with a retrovirus expressing a constitutively active FoxO3a-S207D mutant (FoxO3a-SD), and then transplanted infected bone marrow into lethally irradiated recipient mice. This construct conferred a 3- to 4-fold survival advantage on peripheral *Mst1* ***^−/−^*** CD8^+^ T cells, examined 4 weeks after transplantation, compared to those transplanted with cells transduced with a control pMSCV-IRES-GFP (Mig) virus (Figure 5C; *p* = 0.014). In contrast, transplanted bone marrow cells transduced with a non-phosphorylatable S207A FoxO3a mutant (FoxO3a-SA) were much less effective in rescuing the population of *Mst1* **^−/−^** CD8^+^ T cells than were FoxO3a-SD-transduced cells (Figure 5C; *p* = 0.067). Interestingly, the *Mst1* **^−/−^** CD4^+^ T cell population showed maximum survivability in peripheral blood after transplantation with bone marrow cells transduced with wild-type FoxO3a. The responsiveness of FoxO3a-SD could be different in peripheral CD4^+^ and CD8^+^ T cells in the context of oxidative stress.

### Mst1 Confers ROS Resistance in Lymphocytes

Lymphocytopenia can be induced by a variety of cytotoxic stresses, including chemotherapy and radiation. Radiation generates oxygen-derived free radicals in peripheral lymphocytes [21]. To address the capacity of Mst1 in T cells to protect against irradiation-induced lymphopenia, we examined peripheral T cell viability after exposure to γ-irradiation (5 Gy). Seventy-two hours after γ-irradiation, we found dramatic increases in the viability of peripheral CD4^+^ (∼2.5-fold) and CD8^+^ (∼4-fold) T cells from *Mst1* Tg;*Mst^+/−^* mice compared to *Mst1^+/−^* T cells (Figure 5D, left). Using sublethal doses (5 mg/kg) of N,N'-dimethyl-4,4'-bipyridinium dichloride (paraquat), a herbicide that induces the formation of ROS [22], we found that intracellular ROS levels were dramatically increased in peripheral CD4^+^ and CD8^+^ T cells of *Mst1^+/−^* and *Mst1* **^−/−^** mice within 24 hours (unpublished data). However, overexpression of Mst1 (in *Mst1* Tg;*Mst^+/−^* mice) effectively neutralized intracellular ROS in peripheral T cells in association with approximately a 3.5-fold increase in T cell numbers 6 days after injection (Figure 5D, right).

**Figure 6:**
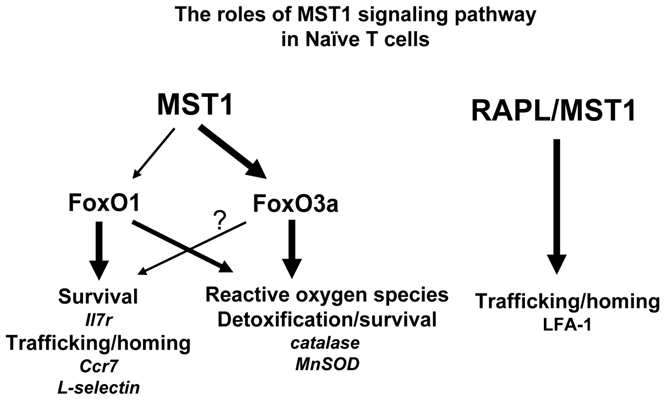
Proposed model of Mst1-FoxO pathway in naïve T cells. In peripheral T cells, Mst1 is activated after TCR ligation, followed by FoxO1/3 activation, which is important for ROS regulation and survival of naïve T cells. Mst1 might also be activated by Rapl through TCR ligation during migration to lymphoid organs, resulting in LFA-1 clustering, as recently reported by others [7], [8]. Arrow size indicates intensity of activation of its target.

These results suggest that Mst1 plays a role in promoting survival of naïve T cells by detoxifying ROS through the Mst1-FoxO-Sod2/catalase pathway (Figure 6). How does regulating intracellular ROS maintain peripheral naïve T cell homeostasis? The peripheral T cell population needs to be maintained at a near-constant number. Naïve T cell maintenance in the periphery depends on the appropriate stimulation of TCR, whereas effector/memory cells can survive in the absence of TCR ligation [23]–[25]. Consistent with recent reports [7], [8], we confirmed that Mst1 is activated by TCR ligation in T cells (Figure S7). Importantly, we observed that FoxO protein levels and phosphorylation of FoxO proteins were reduced in *Mst1^−/−^* T cells, and found that T cells from *Mst1^−/−^* mice exhibited higher levels of apoptosis (Figures 1 and 4C, right). Our findings thus suggest that the Mst1-FoxO-Sod2/catalase pathway might tightly modulate intracellular ROS in CD4^+^ and CD8^+^ T cells to maintain levels optimal for cell survival.

Importantly, two recent studies showed that FoxO1 is critical for the maintenance of naïve T cell homeostasis by controlling the expression of IL-7Rα, L-selectin, and CCR7 [4], [5]. FoxO1-deficient T cells have defects in both survival and homing to secondary lymphoid organs. *Mst1^−/−^* T cells also have defects in homing capacity [7], [9], and the Rap1-Rapl-Mst1 pathway has been previously suggested to play a role in controlling T-cell trafficking [10], [26]. However, unlike FoxO1-deficient T cells, Mst1-deficient T cells appeared to show insignificant changes in the expression of IL-7Rα, L-selectin, CCR7, but Sod2 and catalase were significantly reduced (Figures 2, 3 and S5). It is possible that although the residual levels of FoxO proteins in *Mst1^−/−^* T cells are too low to promote sufficient expression of Sod2 and catalase to protect cells from oxidative damage, they are high enough to maintain expression of other FoxO transcriptional targets involved in T cell trafficking and survival. Thus, increased ROS in *Mst1^−/−^* naïve T cells might also result in a homing defect to secondary lymphoid organs. In addition to the Rap1-Rapl-Mst1 pathway [7], [10], [26], the Mst1-FoxO pathway might also be important for T cell trafficking. Unlike *Mst1* **^−/−^** mice, which show a deprivation of peripheral naïve T cells, Rapl-deficient mice exhibit concentrations of peripheral blood lymphocytes comparable to those of wild-type mice [26]. We also found that Nore1/Rassf5-deficient mice, which are deficient for both Nore1A and Nore1B/Rapl splice variants, exhibited normal numbers of peripheral blood lymphocytes, even though the numbers of T cells in lymph node and spleen were reduced (Figure S8A). It is also reported that *Mst1^−/−^* thymocytes have defects in egress from thymus, which result in lymphopenia in peripheral lymphoid organs [7], [9]. However, we did not observe the accumulation of CD4 or CD8 single positive thymocytes in thymus from *Mst1^−/−^* mice (Figure S1D). It is interesting that Rapl-deficient mice also have defects in thymocyte egress and ratio of CD4 or CD8 single positive thymocytes were increased, but actual cell numbers of these cells and numbers of T cells from peripheral blood were apparently normal [26]. Thus, the Rap1-Rapl-Mst1 pathway could be responsible for controlling the T cell trafficking to secondary lymphoid organs. By controlling intracellular ROS levels, the Mst1-FoxO pathway is likely only important for the survival of peripheral blood naïve T cells, a function that is independent of the Rap1-Rapl-Mst1 pathway. We also found that apoptosis was increased in splenic CD4^+^ and CD8^+^ T cells (Figure S9A), but their ROS levels were not different from those of *Mst1^+/+^* (Figure S9B). However, these cells showed decreased protein levels of FoxO1/3 (Figures 1B and S5), Catalase and Sod2 (Figure S5). Therefore, environmental differences between spleen and peripheral blood might have differentially affected ROS levels in splenic and peripheral blood T cells of *Mst1^−/−^*. In addition, *Mst1^−/−^* CD4^+^ T cells were rescued by FoxO3a wild-type and FoxO3a-S207A mutant but not by FoxO3a-S207D mutant (Figure 4C). Based on these results, we can not exclude the possibility that there might be still other functional mechanisms for regulating T cell survival by FoxO1/3 and/or Mst1 can regulate T cell homeostasis through other unknown factors.

Previous studies in yeast and mammalian cells have shown that Mst1 promotes cell death in response to oxidative stress by phosphorylating histone H2B [27], [28]. In primary neurons, Mst1 enhances cell death by directly activating FoxO1/3 [6], [11]. In sharp contrast, our in vivo mouse study demonstrates for the first time that Mst1 is crucial for cell survival rather than cell death in response to intracellular ROS, at least in naïve T cells, and it accomplishes this function by regulating FoxO proteins. Thus, Mst1 appears to act in a cell context-dependent manner to differentially regulate cell death and survival by modulating FoxO1/3 function. It will be interesting to determine whether the loss of Mst1 promotes cell survival in response to oxidative stresses in primary neurons.

The Hippo signaling pathway restricts cell proliferation and promotes apoptosis in *Drosophila* [29]. Consistent with this, epithelial tissues of *WW45^−/−^* mice also displayed hyperproliferation and defects in apoptosis [30]. Hippo signaling has also been proposed to regulate mammalian organ size [31], but neither *Mst1^−/−^* nor *Mst2^−/−^* mice showed any epithelial hyperplasia or increase in the size of any organs (unpublished data). Notably, *Mst2^−/−^* T cells showed no reduction in the numbers of T cells in peripheral lymphoid organs (Figure S8B), suggesting that Mst2 is dispensable for maintaining naïve T cells homeostasis. In addition, *Mst1^−/−^*;*Mst2^−/−^* mice died at about E8.5 with severe growth retardation, failed placental development, impaired yolks sac and embryo vascular patterning, and increased apoptosis in placentas and embryos [32]. Thus, although Mst1 and Mst2 kinases are functionally similar, both play essential roles in early mouse development. Taken together, these observations suggest that Mst1, but not Mst2, plays a distinct role in maintaining naïve T cells homeostasis.

Finally, we showed that overexpression of Mst1 rescues T cells from apoptosis caused by abnormally elevated intracellular ROS during exposure to anticancer treatments. Thus, Mst1 may represent a potential target of therapeutic strategies to ameliorate lymphopenic side effects in patients undergoing radiation treatment or chemotherapy.

## Materials and Methods

### Ethics Statement

Animal care and experimentation were performed in accordance with procedures approved by the KAIST (Korea Advanced Institute of Science and Technology) Animal Care and Use Committee (KACUC).

### Mice

Generation of *Mst1^−/−^* and *Mst2^−/−^* mice was described in a separate publication [32]. *Nore1^−/−^* mice were obtained from Dr. Sean Lee.

### Generation of Antibodies

An antibody to Mst1 was generated as previously described [30]. An antibody to p-FoxO1 (S212)/p-FoxO3 (S207) was generated by injecting rabbits with the phosphopeptide antigen, C-SAGWKNpSIRHNLS. Antibodies were affinity purified from immune sera using the appropriate antigens.

### Flow Cytometric Analysis

Thymus, spleen, and inguinal lymph nodes were filtered through a 40-µm Cell Strainer (BD Falcon), and the resulting lymphocytes were isolated by centrifugation over Lymphocyte Separation Media (Mediatech Cellgro). Peripheral blood was lysed with Ack lysis buffer (0.15 M NH_4_Cl, 10 mM KHCO_3_, 0.1 mM Na_2_EDTA, pH 7.2) to obtain leukocytes. Isolated cells were stained with antibodies against CD4 (GK1.5), CD8a (53-6.7; Southern Biotech), CD44 (IM7), CCR7 (EBI-1), CD127 (SB/199; eBioscience), and CD62L (MEL-14; BD Pharmingen). Intracellular ROS levels were assessed by prestaining 10^6^ cells with antibodies against CD4 and CD8, followed by staining with DCF-DA (Invitrogen) at a concentration of 20 µM for 30 min at 37°C. Apoptosis was determined in freshly isolated lymphocytes by first prestaining for CD4 and CD8, after which lymphocytes were stained with Annexin V staining kit (BD Pharmingen). The mitochondrial transmembrane potential (ψ_m_) of lymphocytes was determined by staining 10^6^ lymphocytes (prestained for CD4 and CD8) with Rhodamine 123 at a final concentration of 4 µg/µl for 20 min at 37°C. Cells treated with 20 µM CCCP for 20 min before Rhodamine 123 staining were used as a control. Analyses were performed on a triple-laser LSRII flow cytometer with DiVA software (Beckton Dickinson).

### Magnetic-Activated Cell Sorting

Lymphocytes were isolated from spleen and lymph nodes. CD4^+^ and CD8^+^ T cells were purified using CD4 and CD8a microbeads, respectively, and CD4^+^/CD62L^hi^ T cells were purified using CD4^+^CD62L^+^ T Cell Isolation Kit II with a VarioMacs magnetic cell sorter (Miltenyi Biotec).

### Immunoblot Analysis

Magnetic-sorted lymphocytes were lysed in 0.5% NP-40 lysis buffer containing protease and phosphatase inhibitors. Whole-cell lysates were separated by SDS-PAGE on 7.5%–12% gradient gels, transferred to nitrocellulose membranes, and immunoblotted with antibodies against Mst1, FoxO1, FoxO3a (Cell Signaling), and p-FoxO1 (S212)/p-FoxO3 (S207) (Biosource).

### Immunostaining

For intracellular staining, T lymphocytes were prestained for CD4 and CD8, fixed with 4% paraformaldehyde, permeabilized with SAP buffer, and stained with antibodies against FoxO3a and p-FoxO1(S212)/p-FoxO3(S207) (generated by our laboratory). Samples were analyzed by confocal microscopy (LSM510, Carl Zeiss).

### Constructs and Bone Marrow Transplantation

Human FoxO3a-S207D and FoxO3a-S207A cDNAs were generated and cloned into the pMSCV-IRES-GFP retroviral backbone, MIG (Clontech). Retroviruses were produced by transiently transfecting 293T cells with the plasmids together with pEQPAM3 and pVSV-G (Clontech). Retroviruses were transduced into bone marrow mononuclear (BMMN) cells harvested from femurs and tibiae of 4–6-week-old *Mst1^−/−^* mice using “spinfection” [33]. These virally transduced BMMN cells were injected into the tail vein of irradiated (12 Gy total: 7 Gy followed by 5 Gy 3 h later) normal C57BL/6J mice. After 4 wk, blood was collected and GFP-positive cells were analyzed by FACS.

### *In Vitro* NAC Treatment

For *in vitro* NAC experiments, peripheral blood was collected and lysed with Ack lysis buffer containing NAC (10 ng/ml, Sigma). The concentration of NAC was maintained in all reagents used in subsequent steps of DCF-DA staining and Annexin V experiments.

### Quantitative RT-PCR

Total RNA from freshly isolated or magnetically sorted lymphocytes from each organ was isolated using TRIzol Reagent (Intron) according to the manufacturer's instructions and treated with DNaseI. RNA was reverse transcribed using Superscript III (Invitrogen). Quantitative RT-PCR reactions were performed in triplicate using iQ SYBR Green Supermix and the iQ5 Multicolor Real-Time PCR Detection System, and data were analyzed using iQ5 optical system software (Bio-Rad). Gene-specific primer sequences were obtained from Primer Bank (http://pga.mgh.harvard.edu/primerbank). PCR primers used were 5′-CAG ACC TGC CTT ACG ACT ATG G-3′ and 5′-CTC GGT GGC GTT GAG ATT GTT-3′ for Sod2; 5′-AGA GAG CGG ATT CCT GAG AGA-3′ and 5′-ACC TTT CCC TTG GAG TAT CTG G-3′ for catalase; 5′-ATG CCT TTG TGG AAC TAT ATG GC-3′ and 5′-GGT ATG CAC CCA GAG TGA TGC-3′ for Bcl-2; 5′-GAC AGA ACC GCA AGG TAA TCC-3′ and 5′-ACT TGT CAC AAC TCA TGG GTG-3′ for Bim_EL_; and 5′-AGG TCA TCA CTA TTG GCA ACG A-3′ and 5′-CAC TTC ATG ATG GAA TTG AAT GTA GTT-3′ for β-actin.

### Statistics

Statistical analyses of differences between two groups were performed using two-tailed Student's *t-*tests. A *p*-value <0.05 was considered statistically significant.

## Supporting Information

Figure S1 Histology of spleen, lymph nodes, and thymus from control and *Mst1^−/−^* mice. (A) H&E staining of splenic tissue sections. Rp: red pulp; Wp: white pulp. (B) H&E staining of lymph node tissue sections. TZ: T cell zone; BF: B cell follicle. (C) H&E staining of thymus sections. M: medulla; C: cortex. (D) Thymocyte subset numbers quantified from *Mst1^+/+^* (solid bars), *Mst1^+/−^* (grey bars), and *Mst1^−/−^* (open bars) mice by FACS (n≥5). Error bars indicate SEM. (E) Representative FACS profiles of T cell subsets in thymus from *Mst1^+/+^* and *Mst1^−/−^* mice.
(3.80 MB TIF)

Figure S2 Reduced numbers of peripheral T cells in *Mst1^−/−^* mice. (A) Total lymphocytes, CD4^+^ T cells and CD8^+^ T cells in spleen (n≥6), inguinal lymph nodes (n≥4), and peripheral blood (n≥8) from *Mst1^+/+^* (solid bars), *Mst1^+/−^* (grey bars), and *Mst1^−/−^* (open bars) mice (age 6–8 weeks) were quantified. **, *p*<0.01; ***, *p*<0.001; n.s., not significant, compared with *Mst1^+/+^* lymphocytes. (B) NaÃ ^ve (CD62L^hi^CD44^lo^) and effector/memory (CD62L^lo^CD44^hi^) T cell subset numbers in spleen (n≥4), lymph nodes (n≥4), and peripheral blood (n≥6) from *Mst1^+/+^* (solid bars), *Mst1^+/−^* (grey bars) and *Mst1^−/−^* (open bars) mice were quantified by FACS. Error bars indicate SEM. **, *p*<0.01; ***, *p*<0.001, compared with *Mst1^+/+^* lymphocytes.
(0.29 MB TIF)

Figure S3 Inhibition of Fas-FasL interaction in *Mst1^−/−^* mice did not increase the number of peripheral lymphocytes. *Fas^lpr/lpr^* or *FasL^gld/gld^* mice were crossed with *Mst1^−/−^* mice, and CD4^+^ and CD8^+^ T cell numbers in peripheral blood collected from progeny (5-6-weeks old) were quantified. Data show that CD4^+^ or CD8^+^ T cells were not rescued in *Mst1^−/−^*;*Fas^lpr/lpr^* or *Mst1^−/−^*;*FasL^gld/gld^* mice compared to *Mst1^−/−^* mice (n = 3). Error bars indicate SEM.
(0.15 MB TIF)

Figure S4 Analysis of mouse cytokine levels in serum from *Mst1^+/−^* and *Mst1^−/−^* mice. Blood from *Mst1^+/−^* (solid bars) and *Mst1^−/−^* (grey bars) mice collected by tail bleeding was allowed to clot for 2 hours at room temperature before centrifuging for 20 minutes at approximately 2000× g. Relative levels of cytokines and chemokines were determined by assaying sera according to the manufacturer's instruction (Proteome Profiler Array; R&D systems).
(0.67 MB TIF)

Figure S5 Downstream targets of FoxO1/3 are reduced in *Mst1^−/−^* peripheral T cells. Western blot analysis of splenocytes from *Mst1^+/−^*, *Mst1^−/−^* and *Mst1* Tg;*Mst1^−/−^* mice. CD4^+^ and CD8^+^ T cells from spleens were purified by MACS. Cell lysates were then analyzed by immunoblotting. Similar results were obtained from three independent experiments.
(0.45 MB TIF)

Figure S6 γ-H2AX levels in peripheral blood CD4^+^ and CD8^+^ T cells from *Mst1^+/−^* and *Mst1^−/−^* mice. For phospho-Histone H2AX detection, lymphocytes (prestained for CD4 and CD8) were fixed with 4% paraformaldehyde, permeabilized with SAP buffer (0.1% saponin, 0.05% NaN_3_ in Hank's Balanced Salt Solution) and stained with anti-phospho-Histone H2AX-FITC (upstate).
(0.24 MB TIF)

Figure S7 Mst1 is activated after TCR stimulation. Western blot analysis of wild-type T lymphocytes after TCR stimulation. Splenocytes were purified by MACS. Purified CD4^+^ (2×10^6^ cells) or CD8^+^ (1×10^6^ cells) T lymphocytes were cultured on 24-well plates containing pre-bound anti-CD3 or anti-CD3/CD28 (10 ug/ml) antibodies for 24 h. Lysates were separated by SDS-PAGE and immunoblotted for FoxO3a, p-FoxO1/3, MST1, and p-MST1 (T183).
(0.35 MB TIF)

Figure S8 Peripheral T cell subsets from *Nore1^−/−^* and *Mst2^−/−^* mice. (A) Total lymphocytes, CD4^+^ T cells, CD8^+^ T cells, and naÃ ^ve (CD62L^hi^CD44^lo^) and effector/memory (CD62L^lo^CD44^hi^) T cell subset numbers in spleen, inguinal lymph nodes, and peripheral blood were quantified from wild-type (solid bars), and *Nore1^−/−^* (open bars) mice (age 6-8 weeks, n = 3 for each organ from one experiment). (B) T cell subset numbers in spleen, lymph nodes, and peripheral blood from wild-type (solid bars) and *Mst2^−/−^* (open bars) mice (age 6–8 weeks, n≥3 for each organ from two independent experiments) were quantified. Error bars indicate SEM.
(0.26 MB TIF)

Figure S9 Cell death and intracellular ROS levels in *Mst1^−/−^* T cells from spleen. (A) Apoptotic cell death of peripheral blood T cells from *Mst1^+/+^* and *Mst1^−/−^* mice (n≥3) was detected. Relative percentage of Annexin V-positive cells was determined. Error bars indicate SEM. (B) Intracellular ROS levels in splenic CD4^+^ and CD8^+^ T cells from *Mst1^+/+^* and *Mst1^−/−^* mice were detected by staining with DCF-DA (n = 3). Relative FITC-median values of DCF-DA fluorescence were analyzed for CD4^+^ and CD8^+^ populations. Error bars indicate SEM.
(0.16 MB TIF)

## References

[1] Dhein J, Walczak H, Baumler C, Debatin KM, Krammer PH (1995). Autocrine T-cell suicide mediated by APO-1/(Fas/CD95).. Nature.

[2] Hildeman DA, Mitchell T, Teague TK, Henson P, Day BJ (1999). Reactive oxygen species regulate activation-induced T cell apoptosis.. Immunity.

[3] Nakashima I, Suzuki H, Kato M, Akhand AA (2002). Redox control of T-cell death.. Antioxid Redox Signal.

[4] Kerdiles YM, Beisner DR, Tinoco R, Dejean AS, Castrillon DH (2009). Foxo1 links homing and survival of naive T cells by regulating L-selectin, CCR7 and interleukin 7 receptor.. Nat Immunol.

[5] Ouyang W, Beckett O, Flavell RA, Li MO (2009). An essential role of the Forkhead-box transcription factor Foxo1 in control of T cell homeostasis and tolerance.. Immunity.

[6] Lehtinen MK, Yuan Z, Boag PR, Yang Y, Villen J (2006). A conserved MST-FOXO signaling pathway mediates oxidative-stress responses and extends life span.. Cell.

[7] Katagiri K, Katakai T, Ebisuno Y, Ueda Y, Okada T (2009). Mst1 controls lymphocyte trafficking and interstitial motility within lymph nodes.. Embo J.

[8] Zhou D, Medoff BD, Chen L, Li L, Zhang XF (2008). The Nore1B/Mst1 complex restrains antigen receptor-induced proliferation of naive T cells.. Proc Natl Acad Sci U S A.

[9] Dong Y, Du X, Ye J, Han M, Xu T (2009). A Cell-Intrinsic Role for Mst1 in Regulating Thymocyte Egress.. J Immunol.

[10] Katagiri K, Imamura M, Kinashi T (2006). Spatiotemporal regulation of the kinase Mst1 by binding protein RAPL is critical for lymphocyte polarity and adhesion.. Nat Immunol.

[11] Yuan Z, Lehtinen MK, Merlo P, Villen J, Gygi S (2009). Regulation of neuronal cell death by MST1-FOXO1 signaling.. J Biol Chem.

[12] Schluns KS, Kieper WC, Jameson SC, Lefrancois L (2000). Interleukin-7 mediates the homeostasis of naive and memory CD8 T cells in vivo.. Nat Immunol.

[13] Zheng L, Fisher G, Miller RE, Peschon J, Lynch DH (1995). Induction of apoptosis in mature T cells by tumour necrosis factor.. Nature.

[14] Cinar B, Fang PK, Lutchman M, Di Vizio D, Adam RM (2007). The pro-apoptotic kinase Mst1 and its caspase cleavage products are direct inhibitors of Akt1.. Embo J.

[15] Jang SW, Yang SJ, Srinivasan S, Ye K (2007). Akt phosphorylates MstI and prevents its proteolytic activation, blocking FOXO3 phosphorylation and nuclear translocation.. J Biol Chem.

[16] Van Der Heide LP, Hoekman MF, Smidt MP (2004). The ins and outs of FoxO shuttling: mechanisms of FoxO translocation and transcriptional regulation.. Biochem J.

[17] Kops GJ, Dansen TB, Polderman PE, Saarloos I, Wirtz KW (2002). Forkhead transcription factor FOXO3a protects quiescent cells from oxidative stress.. Nature.

[18] Nemoto S, Finkel T (2002). Redox regulation of forkhead proteins through a p66shc-dependent signaling pathway.. Science.

[19] Karanjawala ZE, Murphy N, Hinton DR, Hsieh CL, Lieber MR (2002). Oxygen metabolism causes chromosome breaks and is associated with the neuronal apoptosis observed in DNA double-strand break repair mutants.. Curr Biol.

[20] Wojciechowski S, Tripathi P, Bourdeau T, Acero L, Grimes HL (2007). Bim/Bcl-2 balance is critical for maintaining naive and memory T cell homeostasis.. J Exp Med.

[21] Cemek M, Enginar H, Karaca T, Unak P (2006). In vivo radioprotective effects of Nigella sativa L oil and reduced glutathione against irradiation-induced oxidative injury and number of peripheral blood lymphocytes in rats.. Photochem Photobiol.

[22] Ishii N, Takahashi K, Tomita S, Keino T, Honda S (1990). A methyl viologen-sensitive mutant of the nematode Caenorhabditis elegans.. Mutat Res.

[23] Mueller P, Massner J, Jayachandran R, Combaluzier B, Albrecht I (2008). Regulation of T cell survival through coronin-1-mediated generation of inositol-1,4,5-trisphosphate and calcium mobilization after T cell receptor triggering.. Nat Immunol.

[24] Murali-Krishna K, Lau LL, Sambhara S, Lemonnier F, Altman J (1999). Persistence of memory CD8 T cells in MHC class I-deficient mice.. Science.

[25] Polic B, Kunkel D, Scheffold A, Rajewsky K (2001). How alpha beta T cells deal with induced TCR alpha ablation.. Proc Natl Acad Sci U S A.

[26] Katagiri K, Ohnishi N, Kabashima K, Iyoda T, Takeda N (2004). Crucial functions of the Rap1 effector molecule RAPL in lymphocyte and dendritic cell trafficking.. Nat Immunol.

[27] Ahn SH, Cheung WL, Hsu JY, Diaz RL, Smith MM (2005). Sterile 20 kinase phosphorylates histone H2B at serine 10 during hydrogen peroxide-induced apoptosis in S. cerevisiae.. Cell.

[28] Cheung WL, Ajiro K, Samejima K, Kloc M, Cheung P (2003). Apoptotic phosphorylation of histone H2B is mediated by mammalian sterile twenty kinase.. Cell.

[29] Harvey KF, Pfleger CM, Hariharan IK (2003). The Drosophila Mst ortholog, hippo, restricts growth and cell proliferation and promotes apoptosis.. Cell.

[30] Lee JH, Kim TS, Yang TH, Koo BK, Oh SP (2008). A crucial role of WW45 in developing epithelial tissues in the mouse.. Embo J.

[31] Pan D (2007). Hippo signaling in organ size control.. Genes Dev.

[32] Oh S, Lee D, Kim T, Kim TS, Oh HJ (2009). A Crucial Role for Mst1 and Mst2 Kinases in Early Embryonic Development of the Mouse.. Mol Cell Biol.

[33] Schwaller J, Anastasiadou E, Cain D, Kutok J, Wojiski S (2001). H4(D10S170), a gene frequently rearranged in papillary thyroid carcinoma, is fused to the platelet-derived growth factor receptor beta gene in atypical chronic myeloid leukemia with t(5;10)(q33;q22).. Blood.

