# Supplementary figures and images for: Mst1-FoxO Signaling Protects Naïve T Lymphocytes from Cellular Oxidative Stress in Mice
Source: PLoS One. 2009 Nov 24;4(11):e8011. doi: 10.1371/journal.pone.0008011 (PMC2776980; doi:10.1371/journal.pone.0008011)

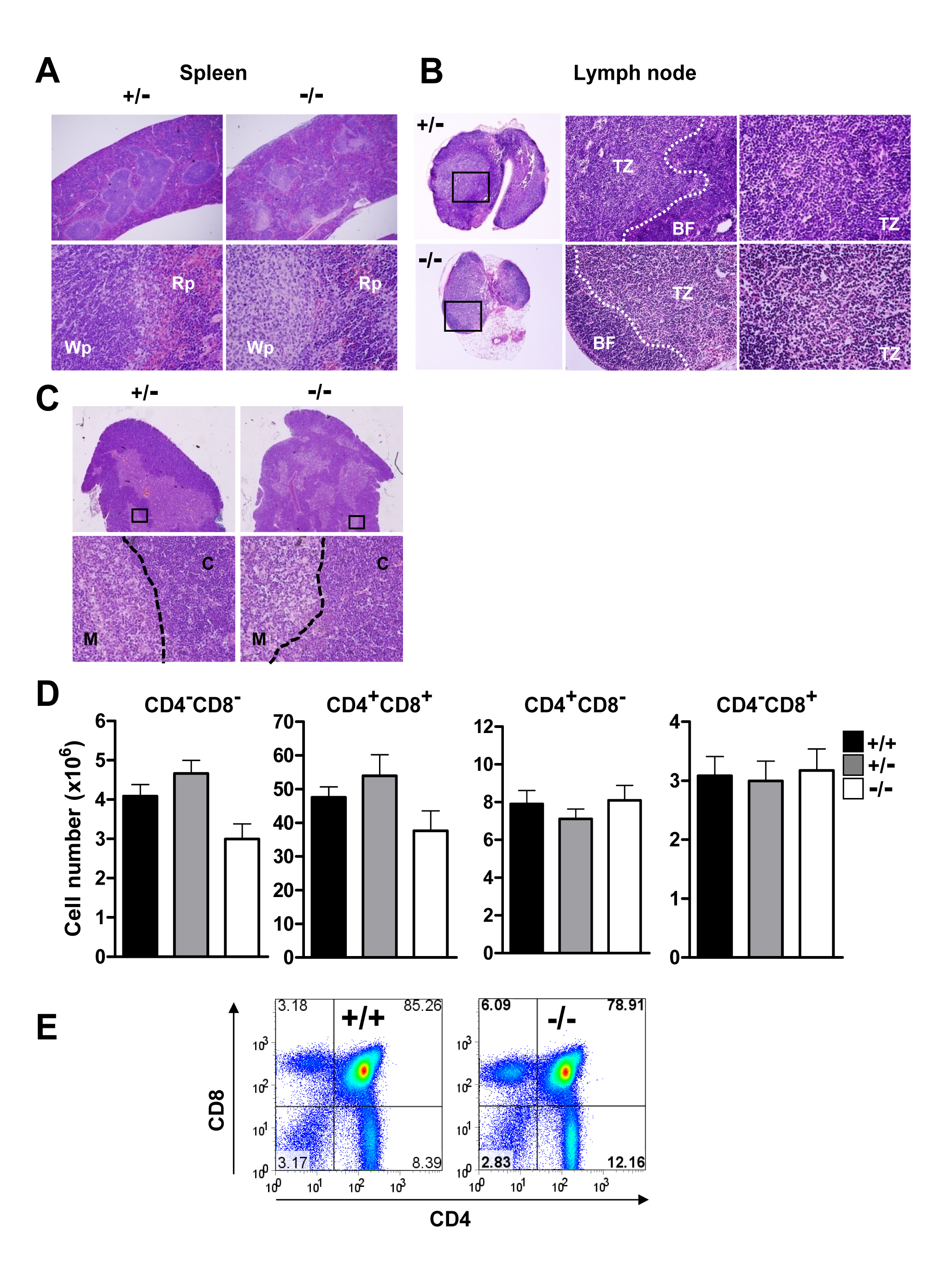

Supplement: Figure S1 — Histology of spleen, lymph nodes, and thymus from control and Mst1−/− mice. (A) H&E staining of splenic tissue sections. Rp: red pulp; Wp: white pulp. (B) H&E staining of lymph node tissue sections. TZ: T cell zone; BF: B cell follicle. (C) H&E staining of thymus sections. M: medulla; C: cortex. (D) Thymocyte subset numbers quantified from Mst1+/+ (solid bars), Mst1+/− (grey bars), and Mst1−/− (open bars) mice by FACS (n≥5). Error bars indicate SEM. (E) Representative FACS profiles of T cell subsets in thymus from Mst1+/+ and Mst1−/− mice. (3.80 MB TIF) [file pone.0008011.s001.tif]

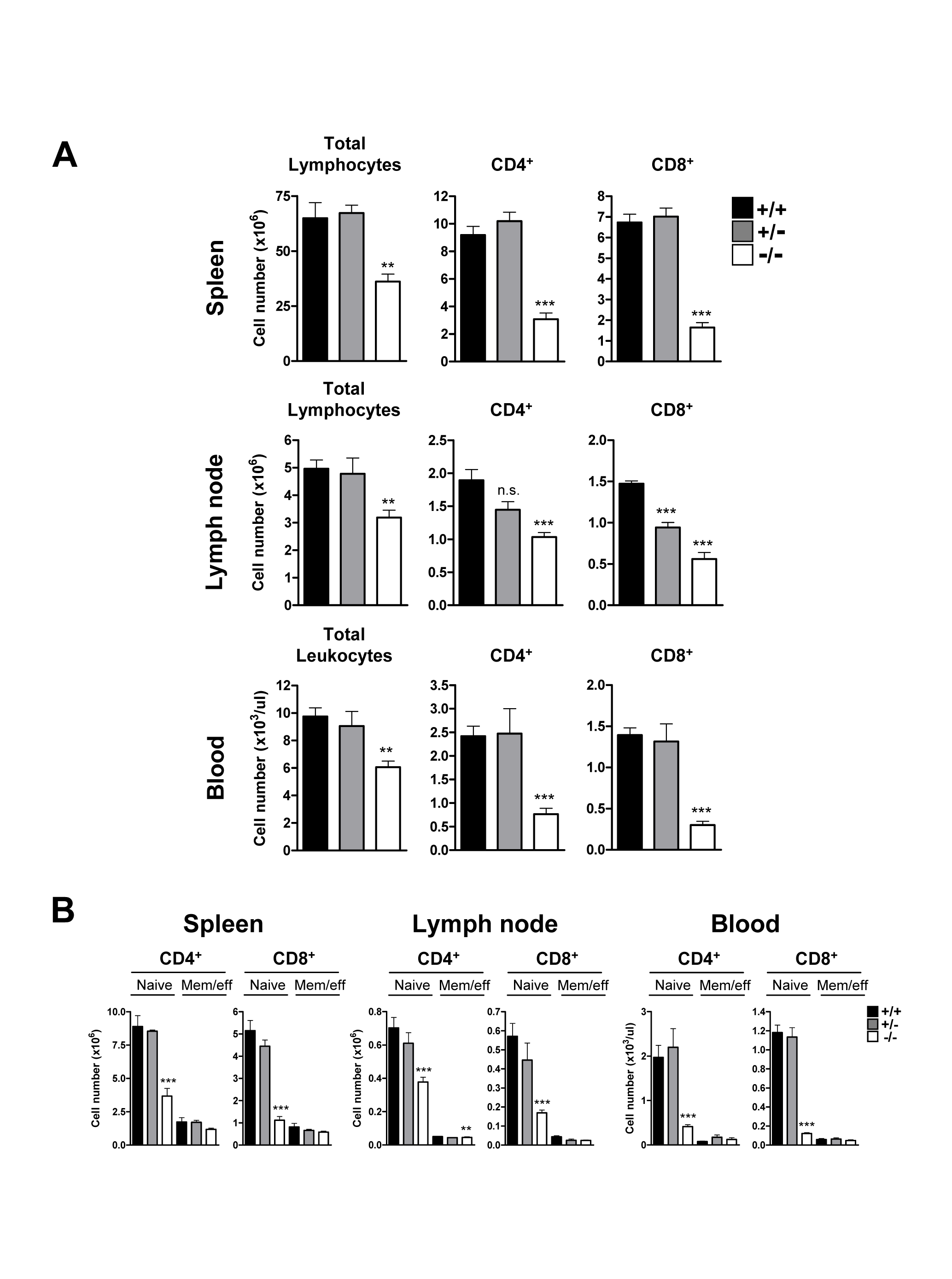

Supplement: Figure S2 — Reduced numbers of peripheral T cells in Mst1−/− mice. (A) Total lymphocytes, CD4+ T cells and CD8+ T cells in spleen (n≥6), inguinal lymph nodes (n≥4), and peripheral blood (n≥8) from Mst1+/+ (solid bars), Mst1+/− (grey bars), and Mst1−/− (open bars) mice (age 6–8 weeks) were quantified. **, p<0.01; ***, p<0.001; n.s., not significant, compared with Mst1+/+ lymphocytes. (B) NaÃ ^ve (CD62LhiCD44lo) and effector/memory (CD62LloCD44hi) T cell subset numbers in spleen (n≥4), lymph nodes (n≥4), and peripheral blood (n≥6) from Mst1+/+ (solid bars), Mst1+/− (grey bars) and Mst1−/− (open bars) mice were quantified by FACS. Error bars indicate SEM. **, p<0.01; ***, p<0.001, compared with Mst1+/+ lymphocytes. (0.29 MB TIF) [file pone.0008011.s002.tif]

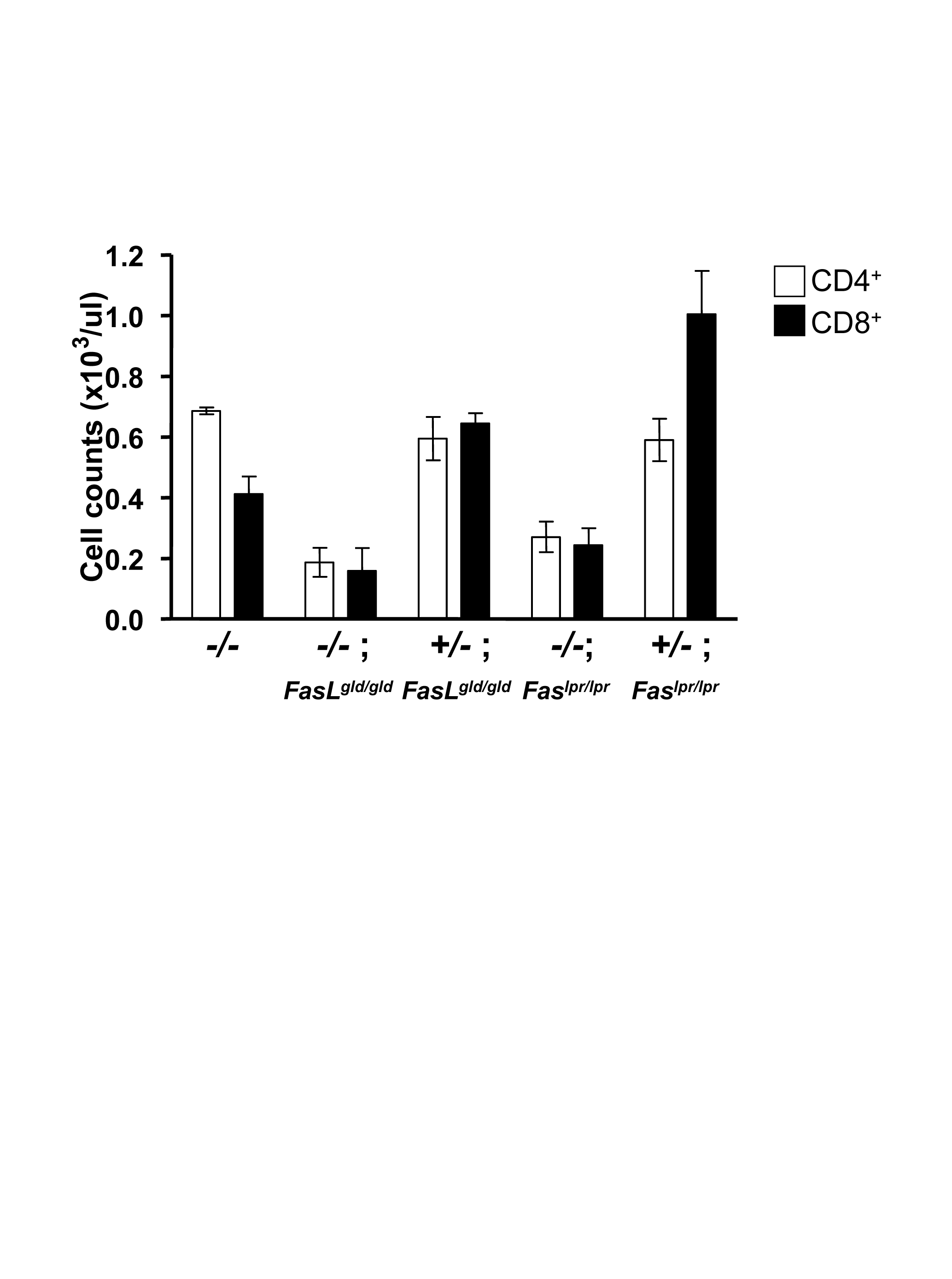

Supplement: Figure S3 — Inhibition of Fas-FasL interaction in Mst1−/− mice did not increase the number of peripheral lymphocytes. Faslpr/lpr or FasLgld/gld mice were crossed with Mst1−/− mice, and CD4+ and CD8+ T cell numbers in peripheral blood collected from progeny (5-6-weeks old) were quantified. Data show that CD4+ or CD8+ T cells were not rescued in Mst1−/−;Faslpr/lpr or Mst1−/−;FasLgld/gld mice compared to Mst1−/− mice (n = 3). Error bars indicate SEM. (0.15 MB TIF) [file pone.0008011.s003.tif]

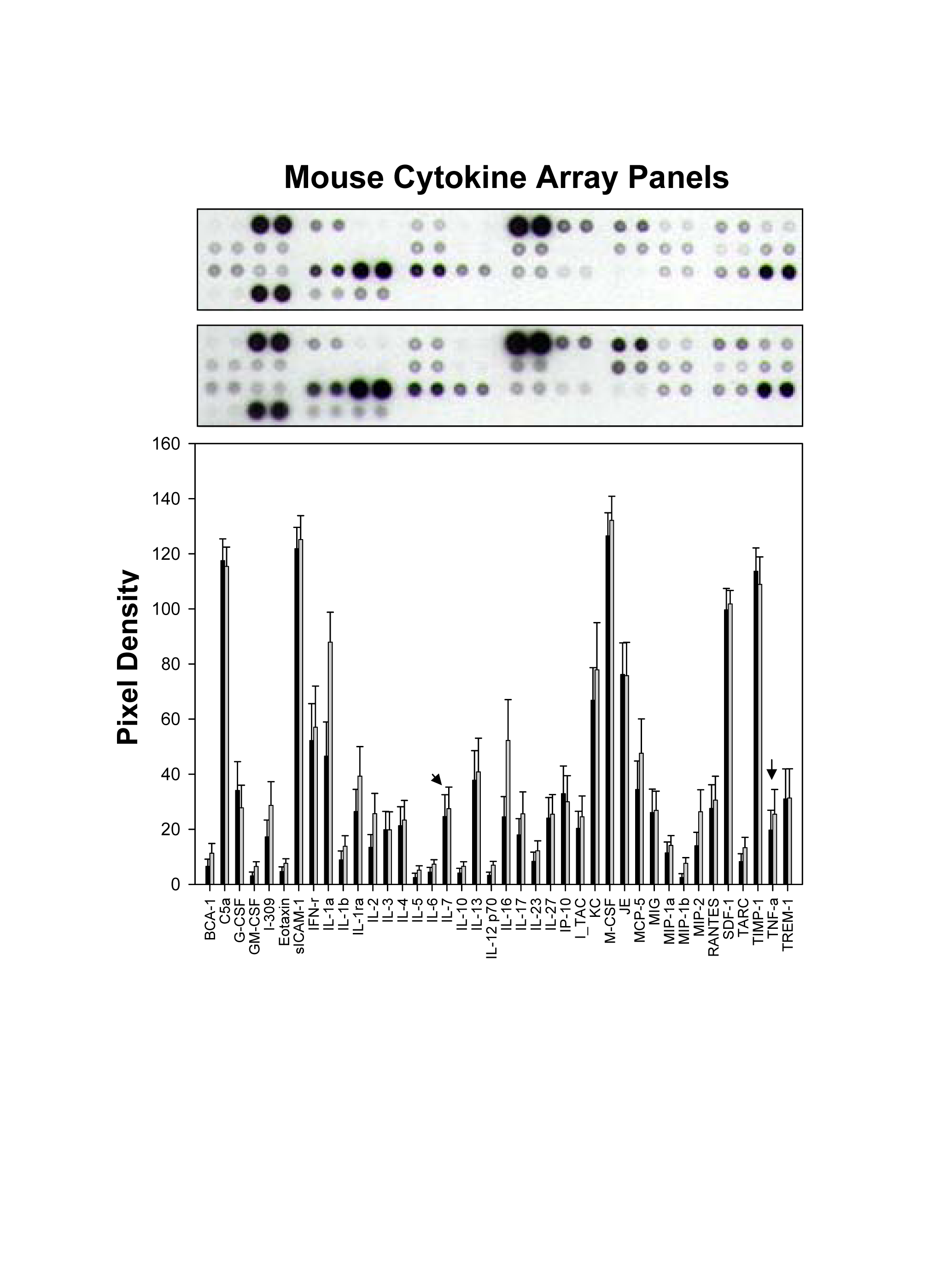

Supplement: Figure S4 — Analysis of mouse cytokine levels in serum from Mst1+/− and Mst1−/− mice. Blood from Mst1+/− (solid bars) and Mst1−/− (grey bars) mice collected by tail bleeding was allowed to clot for 2 hours at room temperature before centrifuging for 20 minutes at approximately 2000× g. Relative levels of cytokines and chemokines were determined by assaying sera according to the manufacturer's instruction (Proteome Profiler Array; R&D systems). (0.67 MB TIF) [file pone.0008011.s004.tif]

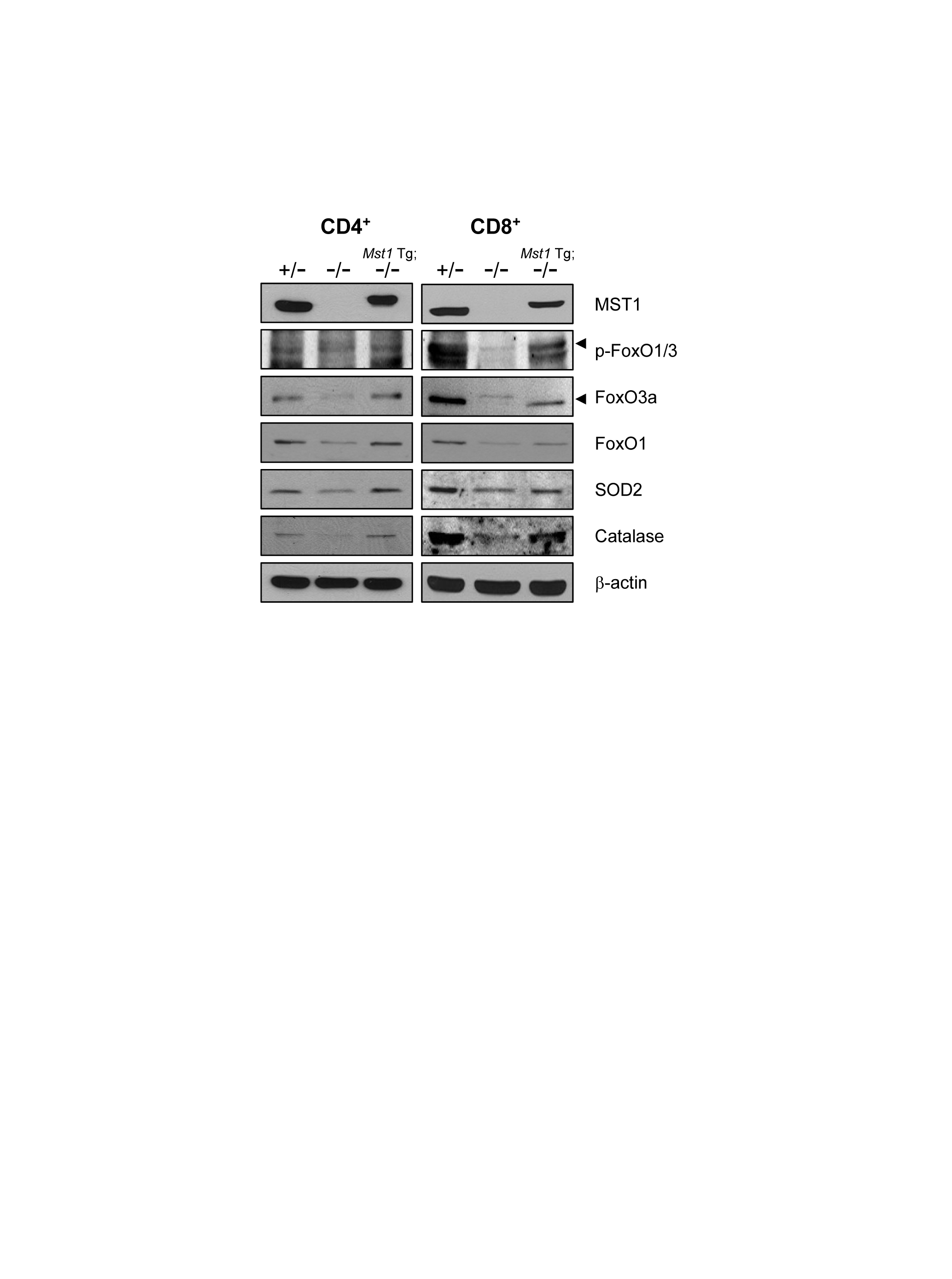

Supplement: Figure S5 — Downstream targets of FoxO1/3 are reduced in Mst1−/− peripheral T cells. Western blot analysis of splenocytes from Mst1+/−, Mst1−/− and Mst1 Tg;Mst1−/− mice. CD4+ and CD8+ T cells from spleens were purified by MACS. Cell lysates were then analyzed by immunoblotting. Similar results were obtained from three independent experiments. (0.45 MB TIF) [file pone.0008011.s005.tif]

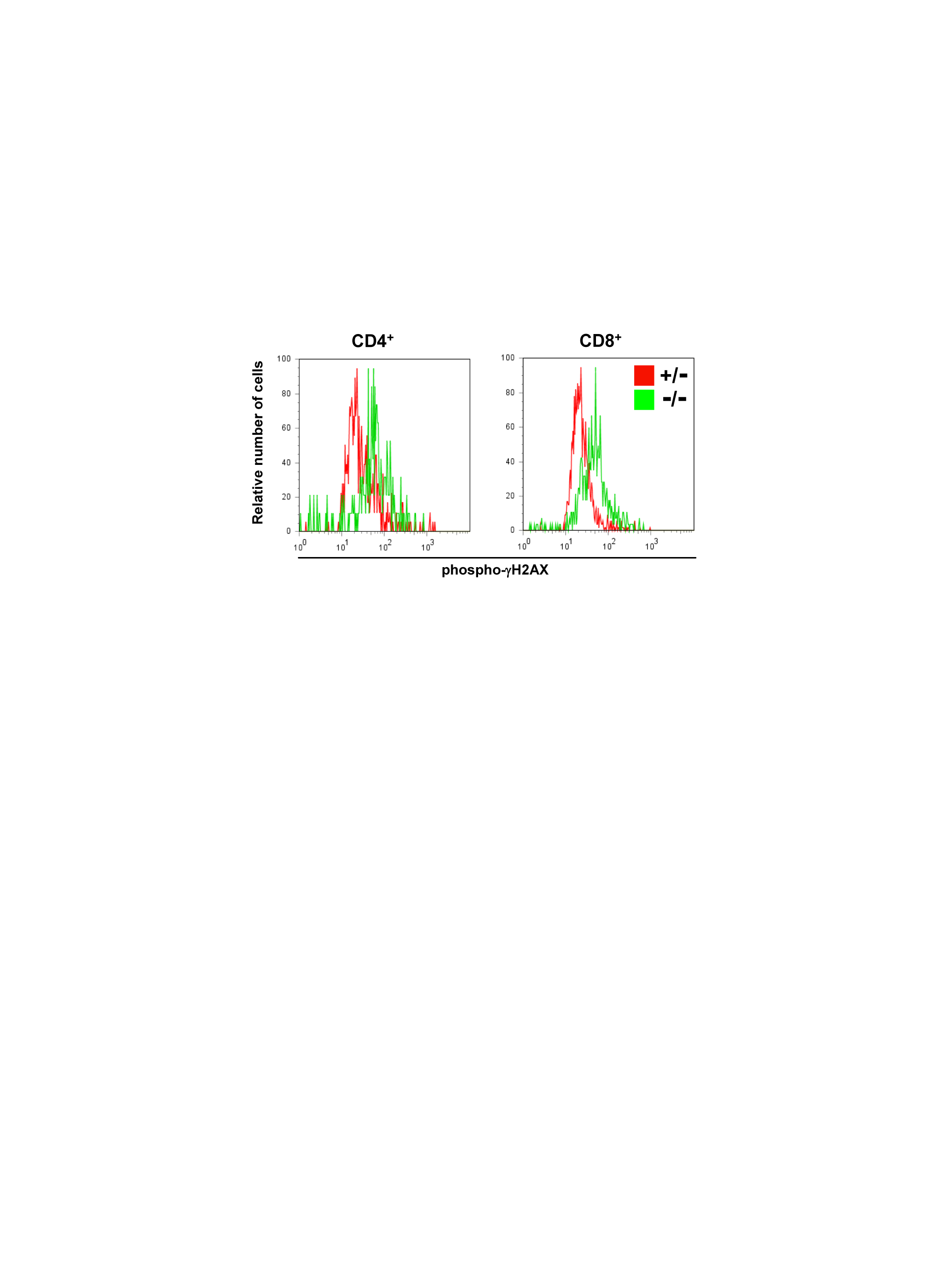

Supplement: Figure S6 — γ-H2AX levels in peripheral blood CD4+ and CD8+ T cells from Mst1+/− and Mst1−/− mice. For phospho-Histone H2AX detection, lymphocytes (prestained for CD4 and CD8) were fixed with 4% paraformaldehyde, permeabilized with SAP buffer (0.1% saponin, 0.05% NaN3 in Hank's Balanced Salt Solution) and stained with anti-phospho-Histone H2AX-FITC (upstate). (0.24 MB TIF) [file pone.0008011.s006.tif]

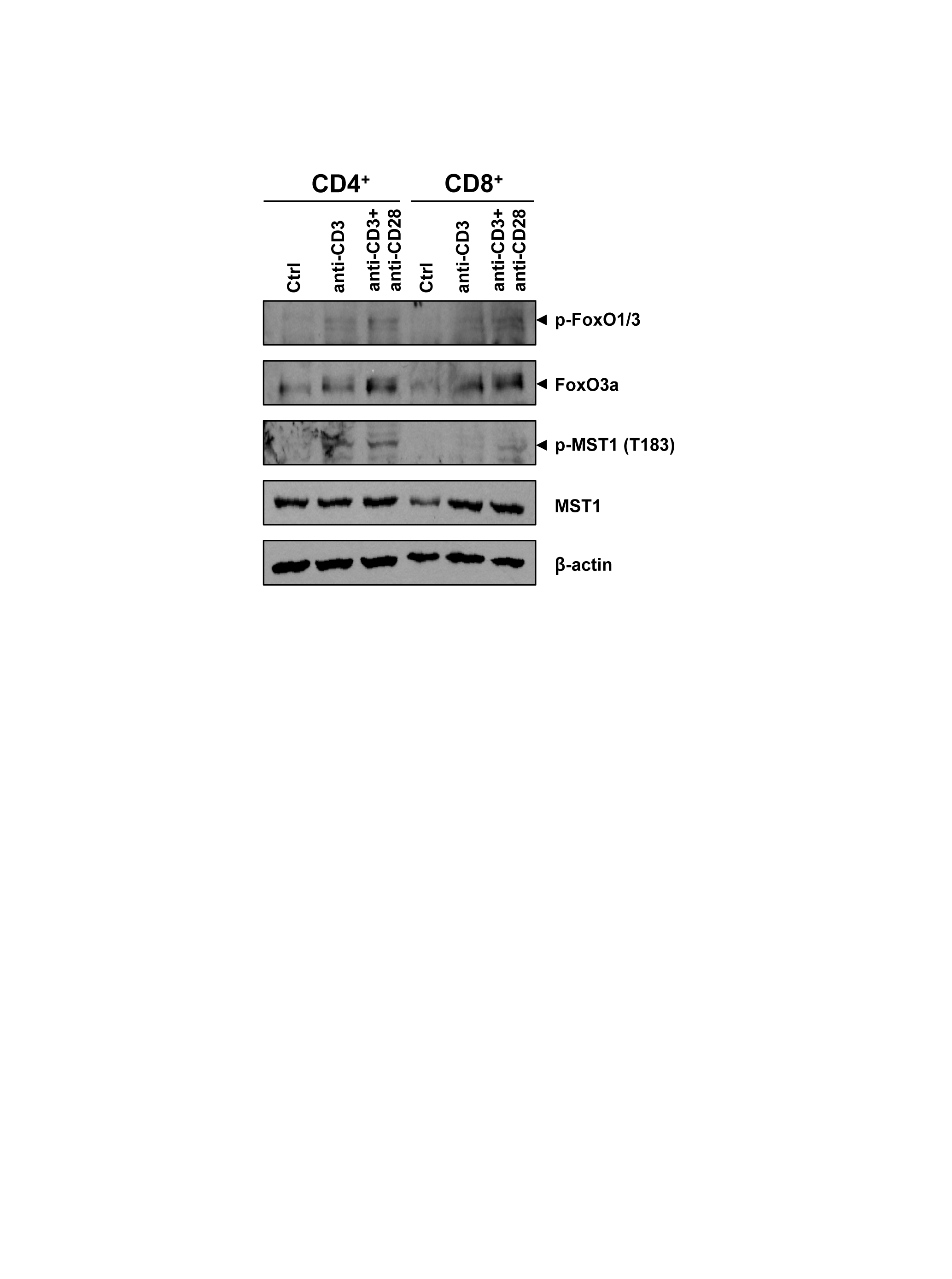

Supplement: Figure S7 — Mst1 is activated after TCR stimulation. Western blot analysis of wild-type T lymphocytes after TCR stimulation. Splenocytes were purified by MACS. Purified CD4+ (2×10^6^ cells) or CD8+ (1×10^6^ cells) T lymphocytes were cultured on 24-well plates containing pre-bound anti-CD3 or anti-CD3/CD28 (10 ug/ml) antibodies for 24 h. Lysates were separated by SDS-PAGE and immunoblotted for FoxO3a, p-FoxO1/3, MST1, and p-MST1 (T183). (0.35 MB TIF) [file pone.0008011.s007.tif]

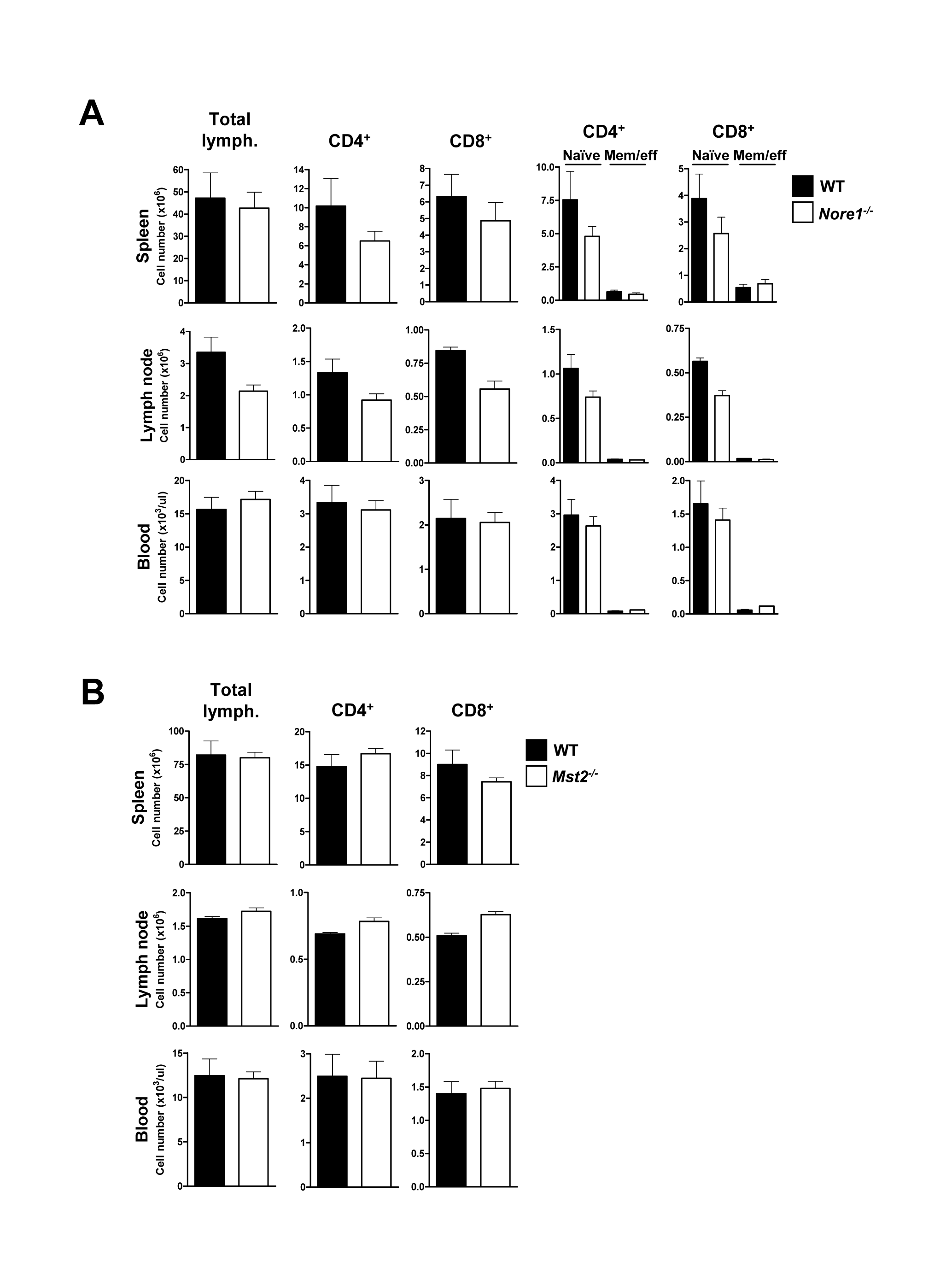

Supplement: Figure S8 — Peripheral T cell subsets from Nore1−/− and Mst2−/− mice. (A) Total lymphocytes, CD4+ T cells, CD8+ T cells, and naÃ ^ve (CD62LhiCD44lo) and effector/memory (CD62LloCD44hi) T cell subset numbers in spleen, inguinal lymph nodes, and peripheral blood were quantified from wild-type (solid bars), and Nore1−/− (open bars) mice (age 6-8 weeks, n = 3 for each organ from one experiment). (B) T cell subset numbers in spleen, lymph nodes, and peripheral blood from wild-type (solid bars) and Mst2−/− (open bars) mice (age 6–8 weeks, n≥3 for each organ from two independent experiments) were quantified. Error bars indicate SEM. (0.26 MB TIF) [file pone.0008011.s008.tif]

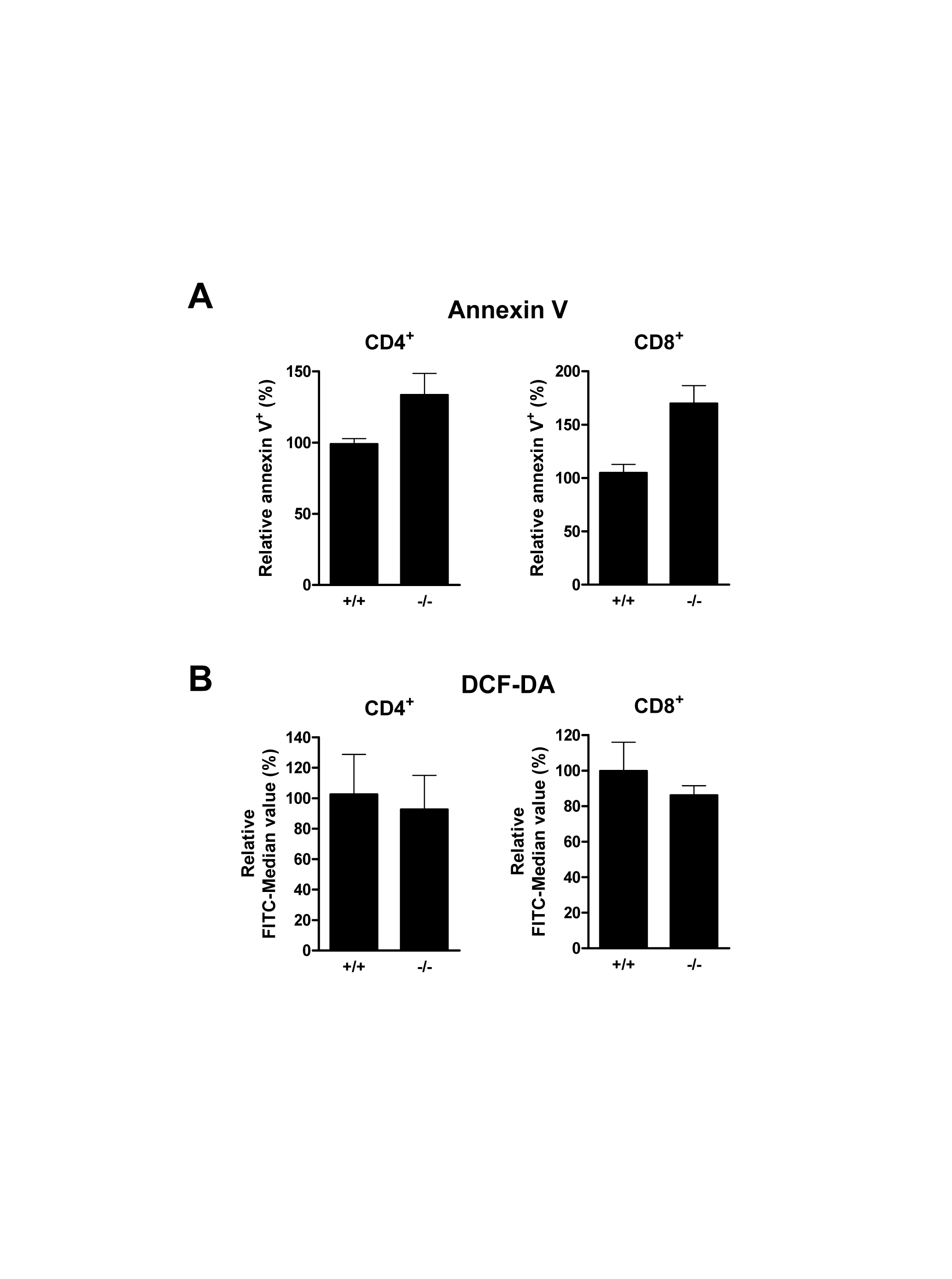

Supplement: Figure S9 — Cell death and intracellular ROS levels in Mst1−/− T cells from spleen. (A) Apoptotic cell death of peripheral blood T cells from Mst1+/+ and Mst1−/− mice (n≥3) was detected. Relative percentage of Annexin V-positive cells was determined. Error bars indicate SEM. (B) Intracellular ROS levels in splenic CD4+ and CD8+ T cells from Mst1+/+ and Mst1−/− mice were detected by staining with DCF-DA (n = 3). Relative FITC-median values of DCF-DA fluorescence were analyzed for CD4+ and CD8+ populations. Error bars indicate SEM. (0.16 MB TIF) [file pone.0008011.s009.tif]
